# *In vivo* dose response and efficacy of the β-lactamase inhibitor, durlobactam, in combination with sulbactam against the *Acinetobacter baumannii-calcoaceticus* complex

**DOI:** 10.1128/aac.00800-23

**Published:** 2023-12-11

**Authors:** John O'Donnell, Angela Tanudra, April Chen, Joseph Newman, Sarah M. McLeod, Rubén Tommasi

**Affiliations:** 1 Entasis Therapeutics Inc., Waltham, Massachusetts, USA; 2 Omega Therapeutics, Watertown, Massachusetts, USA; Providence Portland Medical Center, Portland, Oregon, USA

**Keywords:** durlobactam, sulbactam, pharmacokinetics, pharmacodynamics

## Abstract

Multi-drug resistant (MDR) *Acinetobacter baumannii* is emerging as a pathogen of increasing prevalence and concern. Infections associated with this Gram-negative pathogen are often associated with increased morbidity and mortality and few therapeutic options. The β-lactamase inhibitor sulbactam used commonly in combination with ampicillin demonstrates intrinsic antibacterial activity against *A. baumannii* acting as an inhibitor of PBP1 and PBP3, which participate in cell wall biosynthesis. The production of β-lactamases, particularly class D oxacillinases, however, has limited the utility of sulbactam resorting to increased doses and the need for alternate therapies. Durlobactam is a non-β-lactam β-lactamase inhibitor that demonstrates broad β-lactamase inhibition including class D enzymes produced by *A. baumannii* and has shown potent *in vitro* activity against MDR *A. baumannii*, particularly carbapenem-resistant isolates in susceptibility and pharmacodynamic model systems. The objective of this study is to evaluate the exposure-response relationship of sulbactam and durlobactam in combination using *in vivo* neutropenic thigh and lung models to establish PK/PD exposure magnitudes to project clinically effective doses. Utilizing established PK/PD determinants of %T>MIC and AUC/MIC for sulbactam and durlobactam, respectively, non-linear regressional analysis of drug exposure was evaluated relative to the 24-hour change in bacterial burden (log_10_ CFU/g). Co-modeling of the data across multiple strains exhibiting a broad range of MIC susceptibility suggested net 1-log_10_ CFU/g0 reduction can be achieved when sulbactam T>MIC exceeds 50% of the dosing interval and durlobactam AUC/MIC is 10. These data were ultimately used to support sulbactam-durlobactam dose selection for Phase 3 clinical trials.

## INTRODUCTION

Multidrug-resistant (MDR) *Acinetobacter baumannii* are pathogens increasingly associated with severe nosocomial infections associated with high morbidity and mortality (1 – 5). Previous treatment options are proving to be less effective, and in particular, resistance to carbapenems (1 – 3) has rendered these front-line therapies as no longer viable. Carbapenem-resistant *A. baumannii* (CRAB) has been identified as a global threat with an urgent unmet medical need (6, 7), and recent guidance published by IDSA has recommended high-dose ampicillin-sulbactam (total daily dose of 6–9 g of the sulbactam component) in combination with at least one other agent to treat these carbapenem-resistant strains (8). Sulbactam is a first-generation, narrow spectrum β-lactamase inhibitor used in combination with ampicillin as the marketed product Unasyn. Sulbactam demonstrates intrinsic antibacterial activity against *Acinetobacter* spp. due to its ability to inhibit penicillin binding-proteins 1 and 3 (PBP1 and PBP3) which are essential for cell wall synthesis of Gram-negative bacteria (9) leading to the use of ampicillin-sulbactam in the treatment of *A. baumannii* infections. The higher ampicillin-sulbactam dose recommendation has emerged due to the diminished sulbactam activity against contemporary isolates of *A. baumannii* with the emergence of β-lactamase-mediated resistance (10, 11).

Durlobactam is a non-β-lactam β-lactamase inhibitor with an extended spectrum of activity compared with other β-lactamase inhibitors demonstrating potent inhibition of Ambler class A, C, and D β-lactamases (10), although it has no significant intrinsic antibacterial activity against *A. baumannii* on its own (11). Unique to its activity and ultimate utility to address carbapenem resistance in *A. baumannii*, durlobactam inhibits class D carbapenemases of the OXA family which predominate in these resistant strains. *In vitro* studies have shown that durlobactam protects sulbactam from hydrolysis by β-lactamases and restores its activity against carbapenem-resistant and MDR *A. baumannii* (10). Against 4,038 recent, global, clinical isolates of *A. baumannii*, the addition of durlobactam restored the activity of sulbactam with a shift of the MIC_90_ of sulbactam from 64 µg/mL to 2 µg/mL (12). Recent work utilizing *in vitro* static time kill studies and *in vitro* pharmacodynamic model systems has established exposure targets and PK/PD indices associated the activity of sulbactam and durlobactam with T>MIC in excess of 50% and an AUC/MIC of 10, respectively, associated with achieving a net 1-log_10_ CFU reduction in bacterial burden over 24 hours (13). Utilizing the observed PK/PD indices characterized for sulbactam and durlobactam *in vitro*, the present study seeks to determine exposure magnitudes associated with achieving bactericidal activity *in vivo* using murine neutropenic thigh and lung infection models.

## RESULTS

### *In vitro* susceptibility testing

MICs of sulbactam and sulbactam in the presence of 4 µg/mL of durlobactam are summarized in Table 1 for the sulbactam- and carbapenem-susceptible *A. baumannii* ARC2058 and CRAB isolates used in the neutropenic thigh and lung infection models. A reduction in sulbactam MIC was observed for all strains in the presence of 4 µg/mL of durlobactam, with potentiated MICs (MICs of sulbactam in the presence of 4 µg/mL durlobactam) ranging from 0.5 to 16 µg/mL.

**TABLE 1:** MIC summary of *A. baumannii* isolates used for *in vitro* and *in vivo* PK/PD studies

Isolate	β-Lactamase content	MIC (µg/mL)^*a*^			
		Sulbactam	Sulbactam + durlobactam^*b*^	Meropenem	Murine model^*c*^
ARC2058	ADC-99 [N379S]; OXA-259	2	1	0.5	T, L
ARC3484	ADC-5; TEM-1;OXA-23; OXA-64	16	1	ND^*d*^	T, L
ARC3486	ADC-30; TEM-1; OXA-66; OXA-72	32	0.5	ND	T, L
ARC5077	ADC-82; OXA-72; OXA-66 [K42*]	16	4	ND	T
ARC5079	ADC-214 [T341S]; OXA-65; OXA-72	32	1	ND	T, L
ARC5081	ADC-80; ADC-176; OXA-23; OXA-94	16	4	ND	T, L
ARC5091	ADC-33; OXA-23; OXA-82	8	1	ND	T
ARC5950	ADC-11; OXA-23, OXA-69	64	8	ND	T
ARC5954	ADC-30; TEM-1, OXA-83	32	16	ND	T
ARC5955	ADC-82; TEM-1, OXA-23, OXA-66	64	8	ND	T

^*a*^ Modal MIC.

^*b*^ MIC of sulbactam in the presence of 4 µg/mL durlobactam.

^*c*^ T, thigh model; L, lung model.

^*d*^ ND, not determined.

### *In vivo* neutropenic lung and thigh infection model validation studies

Murine thigh and lung models were validated with meropenem vs. the carbapenem- and sulbactam-susceptible *A. baumannii* ARC2058 isolate with the results summarized in Table 2. Exposure-response analysis of meropenem vs. *A. baumannii* ARC2058 in the neutropenic thigh and lung models are shown in Fig. S1. In the thigh model, the mean meropenem unbound percent time rates above MIC (%*f*T>MIC) associated with a 1-log_10_ CFU/g reduction, 2-log_10_ CFU/g reduction, and the EC80 were 27.2, 32.4, and 36.0, respectively. In the lung model, %*f*T>MIC associated with 1-log_10_ CFU/g reduction, 2-log_10_ CFU/g reduction, and the EC_80_ were 29.4, 32.2, and 39.7, respectively.

**TABLE 2:** Meropenem percentage unbound plasma time above MIC estimates to achieve PK/PD endpoints in neutropenic thigh model vs. *A. baumannii* ARC2058

Compound	Model	PK/PD endpoint		
		CFU reduction from baseline		EC_80_
		1-log_10_	2-log_10_	
Meropenem	Thigh	27.2	32.4	36.0
	Lung	29.4	32.2	39.7

### *In vivo* neutropenic lung and thigh infection model studies with sulbactam alone

In thigh studies with sulbactam alone vs. *A. baumannii* ARC2058, %*f*T>MIC magnitudes associated with 1-log_10_ CFU/g reduction, 2-log_10_ CFU/g reduction, and the EC80 were 20.5, 31.5, and 47.0, respectively (Table 3). In the lung model, the mean %*f*T>MIC magnitudes associated with 1-log_10_ CFU/g reduction, 2-log_10_ CFU/g reduction, and the EC_80_ were 37.8, 50.1, and 68.5, respectively.

**TABLE 3:** Sulbactam percentage unbound plasma time above MIC estimates to achieve PK/PD endpoints alone and in combination with durlobactam in the neutropenic thigh and lung models

Isolate	β-lactamase	Model	PK/PD endpoint		
			CFU reduction from baseline		EC80
			1-log10	2-log10	
ARC2058	ADC-99 [N379S]; OXA-259	Thigh (sulbactam only)	20.5	31.5	47.0
ARC3484	OXA-23;OXA-64;TEM-1	Individual thigh (4:1 dosing)	36.2	40.2	42.8
ARC3486	OXA-66;OXA-72;TEM-1		45.7	52.9	55.3
ARC5079	OXA-65;OXA-72		36.4	42.0	53.2
ARC5081	OXA-23;OXA-66		26.3	28.8	32.0
ARC5091	OXA-23;OXA-78		26.7	29.3	30.4
	Mean ± SD:		34.3 ± 8.1	38.6 ± 10.0	42.7 ± 11.6
ARC2058	ADC-99 [N379S]; OXA-259	Lung (sulbactam only)	37.8	50.1	68.5
ARC3484	OXA-23;OXA-64;TEM-1	Individual lung (4:1 dosing)	58.1	70.9	96.9
ARC3486	OXA-66;OXA-72;TEM-1		45.0	54.5	81.5
ARC5079	OXA-65;OXA-72		41.7	52.8	90.6
ARC5081	OXA-23;OXA-66		37.1	43.9	70.0
	Mean ± SD:		45.5 ± 9.0	55.5 ± 11.3	84.8 ± 11.7
Co-modeled thigh (*n* = 6 strains)			29.9	38.2	44.1
Co-modeled lung (*n* = 5 strains)			41.2	53.5	>100

### *In vivo* neutropenic thigh and lung infection model studies with sulbactam in combination with durlobactam vs. CRAB strains

**Fig 1:**
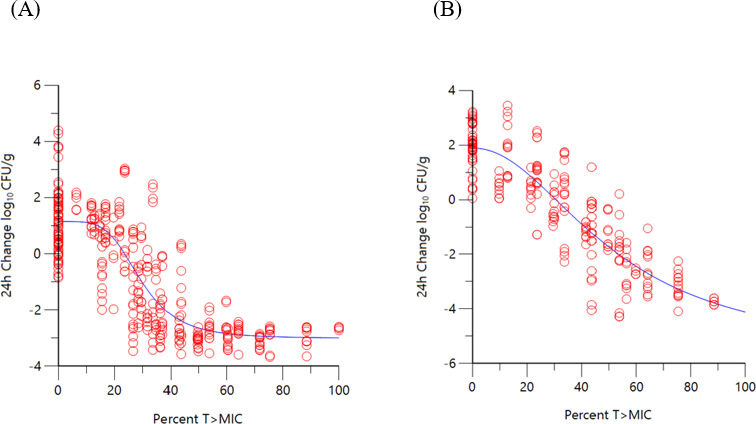
Change in bacterial burden (log_10_ CFU/g) over 24 hours vs. unbound %*f*T>MIC of sulbactam when administered alone and combined with durlobactam in (**A**) neutropenic thigh model (*R* ^2^ = 0.83, *n* = 6 strains) and in (**B**) neutropenic lung model (*R* ^2^ = 0.89, *n* = 5 strains).

Individual strain %*f*T>MIC estimates for sulbactam to achieve PK/PD endpoints of 1-log_10_ CFU/g reduction, 2-log_10_ CFU/g reduction, and the EC_80_ vs. CRAB strains are summarized in Table 3 for thigh and lung infection models utilizing a 4:1 dose titration of sulbactam:durlobactam. Co-modeling of the %*f*T>MIC sulbactam exposure response data (when administered in combination with durlobactam) across multiple CRAB strains and the sulbactam susceptible strain ARC2058 is shown in Fig. 1 for thigh and lung models. Sulbactam %*f*T>MIC magnitudes associated with 1-log_10_ CFU/g reduction, 2-log_10_ CFU/g reduction, and the EC_80_ of the co-modeled data are summarized in Table 3. Magnitudes of %*f*T>MIC were required for 1-log_10_, and 2-log_10_ CFU reduction was nearly identical between the mean of the individual PK/PD endpoints determined across all the strains compared with the PK/PD endpoints determined from co-modeling the data.

**Fig 2:**
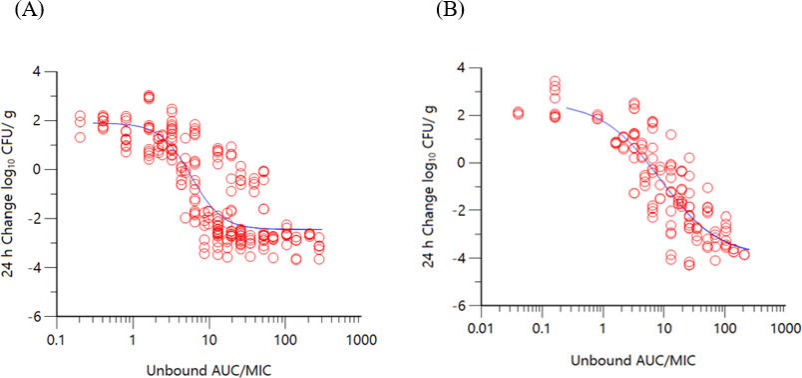
Change in bacterial burden (log_10_ CFU/g) over 24 hours vs. *f*AUC/MIC of durlobactam when administered in combination with sulbactam in (**A**) a neutropenic thigh model (*R* ^2^ = 0.86, *n* = 5 strains) and in (**B**) a neutropenic lung model (*R* ^2^ = 0.91 *n* = 4 strains).

**Fig 3:**
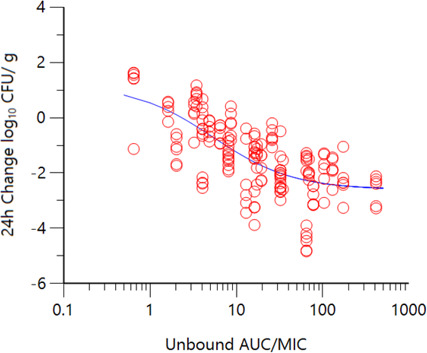
Change in bacterial burden (log_10_ CFU/g) over 24 hours vs. *f*AUC/MIC of durlobactam when administered in combination with sulbactam in a neutropenic thigh model (fixed sulbactam dose, *R* ^2^ = 0.82, *n* = 6 strains).

Co-modeling of the durlobactam exposure response data across multiple MDR *A. baumannii* strains and normalizing the AUCs by the potentiated MIC in the thigh and lung models resulted in Hill-type fits shown in Fig. 2. Correlation coefficients (*R* ^2^) of 0.86 and 0.91 were observed for thigh and lung models, respectively. For studies incorporating a fixed dose of sulbactam to keep %*f*T>MIC constant throughout the durlobactam dose range (Table S1), an *R* ^2^ of 0.82 was observed (Fig. 3). The PK/PD exposure endpoints for durlobactam when sulbactam was administered at a fixed dose are summarized in Table 4. The *f*AUC/MIC magnitudes were generally consistent in both lung and thigh models utilizing a 4:1 dose ratio as well as titrating durlobactam with a fixed dose of sulbactam.

**TABLE 4:** Co-modeled durlobactam *f*AUC/MIC^*a*^ ratio values associated with 1-log_10_ CFU reduction from baseline, 2-log_10_ CFU reduction from baseline, and EC_80_ PK/PD endpoints

Model	# of strains	*R* ^2^	PK/PD endpoint		
			CFU reduction from baseline		EC_80_
			1-log_10_	2-log_10_	
Thigh (4:1 dose)^*b*^	5	0.86	8.0	16.0	15.1
Lung (4:1 dose)^*c*^	4	0.91	10.6	22.4	78.6
Thigh (fixed SUL dose)^*d*^	6	0.82	7.5	31.8	38.2
Mean ± SD			8.7 ± 1.7	23.4 ± 7.9	44.0 ± 32.1

^*a*^ Estimate calculated from Emax fitting of net bacterial CFU change over 24 hours vs. AUC_0-24_ and dividing by MIC of 4.0 µg/mL.

^*b*^ UL mean exposure of 32.9 to 43.5%T>MIC.

^*c*^ UL mean exposure of 43.9 to 81.5%T>MIC.

^*d*^ SUL exposure range of 18.2 to 52.7%T>MIC.

## DISCUSSION

The objectives of the present study were to determine the exposure magnitudes associated with achieving PK/PD endpoints of a net 1-log_10_ and 2-log_10_ CFU reduction over 24 hours in murine neutropenic thigh and lung models. Dose fractionation studies and dose range finding studies performed *in vitro* with sulbactam and durlobactam established the PK/PD indices most closely associated with the activity of each agent (13). Similar to most β-lactams, the time for which unbound concentrations of sulbactam exceed the MIC (%*f*T>MIC) was found to be the index correlated to its activity (14, 15). For durlobactam, unbound *f*AUC/MIC correlated to its activity (13). As observed in the non-linear regression analysis using a Hill-type model with sulbactam and durlobactam exposures, good correlations were observed when co-modeling the data sets across the individual strains. Normalizing the exposure data by MIC, good correlations were observed in Hill-type model fits of the change in log_10_ CFU/g from baseline at 24 hours vs. %*f*T>MIC for sulbactam and *f*AUC/MIC for durlobactam. Convergence of the dose response with the addition of the MIC suggests a proportional relationship between inhibitor exposure and the degree of susceptibility.

Both thigh and lung neutropenic infection models were validated using the carbapenem-susceptible isolate *A. baumannii* ARC2058 and subcutaneous administration of meropenem. It was assumed that the %T>MIC parameter for meropenem best correlates with its activity as this driver has been shown to correlate best with efficacy (16). The magnitude of the EC_80_ for meropenem versus *A. baumannii* ARC2058 matched the EC_80_ for meropenem versus *P. aeruginosa* in the same model (17), as well as the projected magnitude of 40%T>MIC proposed for treating critically ill patient populations (18). For sulbactam treatment versus *A. baumannii* ARC2058, %*f*T>MIC magnitudes required for bactericidal activity (>1-log_10_ CFU reduction over 24 hours) were consistent with the estimated clinical exposures of sulbactam used to successfully treat *A. baumannii* infections based upon doses and MICs observed in human clinical studies (19 – 22) and human PK parameters for sulbactam (23, 24) to determine the PK/PD magnitudes associated with sulbactam efficacy in the clinic. An endpoint of stasis (no net reduction of log_10_ CFU burden over 24 hours) was not considered in the exposure-response analysis as bactericidal PK/PD endpoints of 1- and 2-log_10_ CFU reduction over 24 hours would be more relevant for patients with these serious infections. For MDR *A. baumannii* isolates, exposure response data were obtained from thigh and lung studies performed in mice administered a 4:1 dose ratio of sulbactam:durlobactam. The rationale for the 4:1 dose ratio was to match exposures associated with achieving the required T>MIC and AUC/MIC requirements for sulbactam and durlobactam, respectively, that were associated with bactericidal activity within *in vitro* pharmacodynamic models (13). These exposures corresponded to sulbactam *f*T>MIC greater than ~70% and durlobactam AUC/MIC > 10 which were shown to correlate to at least 1-log_10_ CFU/mL reduction in 24 hours within the *in vitro* models. By comparison, in the present *in vivo* study, a 2-log_10_ CFU reduction was achieved when sulbactam exceeded 50% *f*T>MIC vs. the sulbactam-susceptible *A. baumannii* isolate ARC2058. One weakness inherent in the study design using a titration of a fixed 4:1 dose of sulbactam:durlobactam was that both component exposures varied relative to the MIC across the dose range; however, %*f*T>MIC magnitudes were similar when sulbactam was evaluated vs. ARC2058 and when sulbactam was evaluated vs. CRAB isolates in the presence of durlobactam (Table 3). This consistency in sulbactam exposure targets in susceptible vs. CRAB strains suggests there was sufficient exposure of durlobactam to restore the activity of sulbactam. Magnitudes observed *in vivo* were slightly lower than targeted with %*f*T>MIC of 20.5 and 31.5 for 1- and 2-log_10_ CFU/g reduction, respectively, in the neutropenic thigh model and 31.5 and 50.1 for 1- and 2-log_10_ CFU/g reduction, respectively, in the lung model vs. the sulbactam-susceptible strain ARC2058. Exposure magnitudes were generally higher in the lung vs. thigh infection model. Attempts to target sulbactam exposure at a set duration above the MIC for six MDR strains evaluated in the thigh model were challenging given the variability encountered with establishing a modal MIC for these strains. One-dilution differences in microbroth MIC determinations (which are within the error of the MIC assay) combined with PK variability can lead to broad ranges of values observed for PK/PD endpoints such as %*f*T>MIC or AUC_0-24_/MIC ratio (25, 26). Nevertheless, dose titration of durlobactam exposure with sulbactam at %*f*T>MIC exposures ranging from 18.3 to 43.7% showed *f*AUC/MIC_0-24_ ratios of ~10 and ~30 for 1-log_10_ and 2-log_10_ CFU reductions from baseline at 24 hours, respectively, in the thigh model as well as in other *in vivo* and *in vitro* experiments (13). Interestingly, strains such as *A. baumannii* ARC5950 and ARC5955 harboring higher SUL-DUR MICs of 8 µg/mL, presumably due to mutations to PBP3 had similar sulbactam %*f*T>MIC requirements as observed *in vitro* (13). Although PK/PD exposures were considered in unbound plasma, both sulbactam and durlobactam demonstrated excellent distribution into lung epithelial lining fluid (ELF) of mice with penetration rates of 32% and 63%, respectively Table S2. These values differed from what has been observed clinically (27) where sulbactam and durlobactam penetration was determined to be 50% and 37%, respectively, based on total ELF and plasma AUC exposures. Based on this observation only unbound plasma PK/PD targets were considered for probability of target attainment analysis and Phase 3 dose justification.

In conclusion, sulbactam demonstrated robust bactericidal activity when administered in combination with durlobactam to treat infections caused by CRAB isolates evaluated in murine neutropenic thigh and lung infection models. Using PK/PD indices established from *in vitro* pharmacodynamic model systems, %T>MIC and AUC_0-24_/MIC was highly correlated to the *in vivo* activity of sulbactam and durlobactam, respectively. Both murine neutropenic thigh and lung models suggested a 1-log_10_ CFU reduction over 24 hours can be realized when sulbactam *f*T>MIC is greater than 50% and durlobactam *f*AUC/MIC_0-24_ is ~10. Exposure requirements were slightly higher in the lung model vs. the thigh and were therefore ultimately used for the exposure targets. An additional log_10_ CFU reduction can be realized when durlobactam *f*AUC/MIC_0-24_ is ~30. These targets were used to support dose selection for the Phase 3 clinical trial.

## MATERIALS AND METHODS

### Bacterial isolates

All strains with an “ARC” designation are part of the Entasis collection of clinical isolates. All isolates have been previously characterized by whole genome sequencing.

### Antimicrobial susceptibility testing

Sulbactam and sulbactam-durlobactam broth MIC testing was performed according to the Clinical and Laboratory Standards Institute methodology (28). Sulbactam-durlobactam MIC testing was performed as a titration of sulbactam in the presence of a fixed concentration of 4 µg/mL durlobactam.

### Animal welfare statement

All *in vivo* procedures were completed in compliance with the Animal Welfare Act Regulations (9 CFR 3) under Entasis Therapeutics-reviewed Institutional Animal Care & Use Committee (IACUC)-approved protocols and under the supervision of a site-attending veterinarian.

### *In vivo* neutropenic lung and thigh infection models

*In vivo* infection models of pneumonia and thigh tissue abscess were performed in CD-1 mice (15–18 g) rendered neutropenic by cyclophosphamide treatment prior to infection as previously described (29). In the lung model, mice were infected with *A. baumannii* isolates in 1% agar slurry via direct intratracheal instillation. In the thigh model, mice were infected intramuscularly in the right thigh. Infected mice were treated with either sulbactam alone or sulbactam combined with durlobactam at a constant 4:1 ratio. Dosing was initiated 2 hours after infection and administered as eight subcutaneous (SC) injections administered q3h or four SC injections administered q6h. Vehicle groups received a single SC injection, and positive control groups received either a single SC injection of colistin sulphate (maximum tolerated dose of 40 mg/kg) or two oral doses of levofloxacin (200 mg/kg bid) 2 hours after infection. Colistin sulphate administered as a single 40-mg/kg dose achieved 0.25 to 2.6 log_10_ CFU/g reduction vs. MDR *A. baumannii* strains, and levofloxacin achieved −2.4 log_10_ CFU vs. *A. baumannii* ARC2058 in the thigh model. In the lung model, colistin administration resulted in 1-log_10_ CFU/g growth to 2.1 log_10_ CFU/g reduction vs. MDR *A. baumannii* strains and levofloxacin administration resulted in 1.8 log_10_ CFU/g reduction vs. *A. baumannii* ARC2058. The overall performance of positive controls was acceptable relative to isolate susceptibility. Efficacy was determined by harvesting infected tissue and determining viable bacterial counts 24 hours after the start of treatment. A full dose response study utilized meropenem to validate the model system, where meropenem was administered SC on a q6h regimen from 1 to 300 mg/kg following uranyl nitrate pre-treatment (30) to extend drug exposure. Subcutaneous doses of sulbactam and durlobactam were administered at a 4:1 ratio at doses of 20:5, 40:10, 60:15, 80:20, and 160:40 mg/kg (SUL:DUR) with plasma PK sampling (*n* = 3 samples/timepoint) at 0.5, 1, 2, 3, 4, 6, 8, 10, and 12 hours post dose. These studies were completed in neutropenic CD-1 mice with active thigh infections. An additional PK and ELF distribution study was completed at a SUL:DUR dose of 100:25 mg/kg in neutropenic lung-infected animals with PK timepoints sampled at 0, 1, 3, 6, and 12 hours post dose. Blood was processed for plasma using microcontainer tubes containing ethylenediamine tetraacetic acid (EDTA, Beckton Dickenson) and centrifugation for 5 minutes at 13,200 rpm. Plasma samples were treated 1:1 with a SigmaFAST protease inhibitor cocktail (Sigma-Aldrich, Cat No. S8820) prepared from one tablet dissolved in 10 mL of deionized water. The samples were then stored at −80°C until bioanalysis. Plasma samples were analyzed by LC-MS/MS using instrument parameters summarized in (Table S3)

A murine unbound fraction of 100% was used to calculate free plasma concentrations for both sulbactam and durlobactam used in exposure response analyses. This value came from the mean of the mouse plasma protein unbound fractions determined *ex vivo* at a range of 0.4 µM to 50 µM (0.093 to 11.7 µg/mL for sulbactam and 0.111 to 13.9 µg/mL for durlobactam; data on file Entasis Therapeutics).

### Pharmacokinetic-pharmacodynamic analysis

As the plasma PK of neutropenic lung-infected animals matched that of thigh-infected animals, the thigh-infected PK was used for the development of the population PK model and subsequent exposure estimates for doses used in the PK/PD exposure-response analyses. Pharmacokinetic models were fit to time vs. drug concentration profiles generated in infected mice using Phoenix6.2 WinNonLin and NLME. For the purposes of estimating exposures across all doses used in dose response studies, population PK models were developed for sulbactam alone, for sulbactam when co-administered with durlobactam, and for durlobactam co-administered with sulbactam (dose ratio 4:1, sulbactam:durlobactam). Population PK parameter estimates were derived from a two-compartment PK model incorporating a log-additive error model constructed from fitting of sulbactam and durlobactam concentration vs. time data (Table S4). Representative model fit concentration vs. time profiles vs. observed data are shown in Fig. S1 for sulbactam at 320 and 40 mg/kg and durlobactam at 80 and 10 mg/kg. Predicted exposures were utilized in the PK/PD analyses as all dosing solutions tested were within the variability of the bioanalytical method (20%).

For pharmacokinetic-pharmacodynamic evaluation sulbactam %*f*T>MIC and durlobactam *f*AUC_0-24_/MIC were simulated for each dose using the population PK model. A Hill-type model was fit to the pharmacodynamic data generated from the dose response studies with linear least squares regression analysis of the relationships between change in log_10_ CFU from baseline at 24 hours and sulbactam %*f*T>MIC and durlobactam *f*AUC/MIC. Magnitudes associated with 1-log_10_ and 2-log_10_ CFU reduction from baseline at 24 hours as well as the exposure required for an 80% reduction in bacterial burden (EC_80_) were determined from the fitted function.

### Distribution of sulbactam and durlobactam into mouse ELF

ELF penetration was studied for both sulbactam alone and sulbactam:durlobactam, where mice received a single subcutaneous injection of either 100 mg/kg sulbactam or a combination of 100 mg/kg sulbactam/25 mg/kg durlobactam. Administration was initiated 2 hours after intratracheal infection with *A. baumannii* ARC2058 in neutropenic mice. Whole blood samples were taken by cardiac puncture. ELF was collected immediately following blood collection via bronchoalveolar lavage (BAL), which was performed by inserting a catheter into the trachea and instilling 1 aliquot of 0.8 mL normal saline followed by immediate removal of the fluid. Four mice per time point were used. Plasma and BAL were separated using microcontainer tubes containing EDTA (Beckton Dickenson) by centrifugation for 5 minutes at 13,200 rpm, treated with 1:1 SigmaFAST protease inhibitor cocktail, and stored at −80°C until bio-analysis. The pre-diluted concentration of antibiotic in the BAL fluid was calculated by comparing the difference in amounts of urea measured (Bioassay Systems QuantiChrom Urea Assay Kit) in the simultaneously collected plasma and BAL fluid for each mouse.

### Sulbactam and durlobactam concentration determination

Serial blood samples collected mice infected over a 24-hour time and administered doses from 50:12.5 mg/kg to 320:80 mg/kg sulbactam durlobactam were assayed by liquid chromatography - tandem mass spectrometry (LC-MS/MS) (Sciex API 5000) using a qualified non-GLP bioanalytical method (Table S3). Plasma samples were treated 1:1 with a SigmaFAST protease inhibitor cocktail (Sigma-Aldrich, Cat No. S8820) prepared from one tablet dissolved in 10 mL of deionized water. The samples were then stored at −80°C until bioanalysis. Prepared calibration standards in the assay (1, 2.5, 5, 10, 25, 100, 500, 1,000, 5,000, and 10,000 ng/mL) were made by serial dilution in 1:1 Mueller Hinton Broth 2:analytical sample. The analytical sample consisted of blank plasma or BAL diluted 1:1 in SigmaFAST prior to processing. Sample aliquots (50 µL) were added in the 96-well plates. Then, 150 µL of 100% acetonitrile and 0.1% formic acid containing 250 ng/mL of carbutamide as an internal standard was added to the plate. Plates were covered and centrifuged at 4,000 × *g* for 5 minutes. Supernatant (100 µL) was transferred to a clean collection plate. Samples were mixed well and analyzed by LC-MS/MS (Sciex API 5000—Analyst v1.6.1), and LC-MS/MS instrument parameters are summarized in Table S3. The assay had a lower limit of quantitation (LLOQ) of 1 ng/mL and a upper limit of quantitation (ULOQ) of 10,000 ng/mL. The assay performance is summarized in Table S5.

## SUPPLEMENTAL MATERIAL

Supplemental information aac.00800-23-s0001.docx Cited supplemental tables and figures.

ASM does not own the copyrights to Supplemental Material that may be linked to, or accessed through, an article. The authors have granted ASM a non-exclusive, world-wide license to publish the Supplemental Material files. Please contact the corresponding author directly for reuse.
