# Supplementary material for: In vivo dose response and efficacy of the β-lactamase inhibitor, durlobactam, in combination with sulbactam against the Acinetobacter baumannii-calcoaceticus complex
Source: Antimicrob Agents Chemother. 2023 Dec 11;68(1):e00800-23. doi: 10.1128/aac.00800-23 (PMC10777848; doi:10.1128/aac.00800-23)
Supplement: Supplemental information — Cited supplemental tables and figures. [file aac.00800-23-s0001.docx]

**Supplemental Information**

| **Supplemental Information Table 1. Sulbactam dose and percent unbound plasma Time above MIC estimates when durlobactam is administered with sulbactam at a constant dose** | | | | |
| --- | --- | --- | --- | --- |
| **Isolate** | **β-lactamase** | **SUL-DUR MIC**  **(µg/mL)** | **SUL dose**  **(mg/kg, q3h)** | **SUL**  ***f*%T>MIC** |
| ARC3486 | OXA-66;OXA-72;TEM-1 | 0.5 | 15 | 43.7 |
| ARC5077 | OXA-72 | 4 | 150 | 52.7 |
| ARC5081 | OXA-23;OXA-66 | 4 | 15 | 18.3 |
| ARC5950 | OXA-23;OXA-10; OXA-69 | 8 | 150 | 42.7 |
| ARC5954 | TEM-1,OXA-83;ADC-30 | 16 | 75 | 26.8 |
| ARC5955 | TEM-1, OXA-23,OXA-66, ADC-82 | 8 | 75 | 32.8 |

**Supplemental Information Table 2. Plasma and ELF exposures of 100:25 mg/kg sulbactam:durlobactam in neutropenic mice infected with *A. baumannii* ARC2058**

|  | **Sulbactam Concentration**  **(µg/mL)** | | **Durlobactam Concentration (µg/mL)** | |
| --- | --- | --- | --- | --- |
| **Timepoint (h)** | **Plasma** | **ELF** | **Plasma** | **ELF** |
| 1 | 18.55±5.87 | 5.70±1.15 | 4.07±1.79 | 2.48±0.91 |
| 3 | 1.69±0.56 | 0.54±0.12 | 0.33±0.13 | 0.26±0.15 |
| 6 | 0.10±0.05 | 0.29±0.14 | 0.03±0.01 | 0.002±0.001 |
| 12 | 0.02±0.01 | 0.06±0.01 | 0.01±0.001 | BLQ |
| AUC (µg.h/mL) | 32.3 | 10.3 | 7.0 | 4.4 |
| AUC_ELF_/AUC_plasma_ |  | 0.32 |  | 0.63 |

**Supplemental Information Table 3. UPLC/MS/MS Instrument Conditions for Sulbactam and Durlobactam Quantitation**

| **Instrument** | Schimadzu UPLC - Sciex 5000 LC-MS/MS Mass Spectrometer | |
| --- | --- | --- |
| **Column** | Atlantis T3, 5µ, 50 x 3.0mm | |
| **Column Temperature** | 35^0^C | |
| **Sample Temperature** | 10^0^C | |
| **Flow rate** | 1.200 mL/min | |
| **Gradient** | Time (min) | %B |
|  | Initial | 2.0 |
|  | 0.3 | 2.0 |
|  | 1.3 | 98 |
|  | 1.75 | 98 |
|  | 1.76 | 2.0 |
|  | 2.00 | Stop |
|  |  | |
| **Divert Valve** | 0.30 min to Mass Spec  1.80 min to waste | |
| **Mobile Phase A** | 0.1% formic acid in water | |
| **Mobile Phase B** | 0.1% formic acid in Acetonitrile | |
| **MRM** | Source Type Turbo Spray  Polarity: Negative (Positive for Meropenem and Imipenem)  Resolution Q1: Unit  Resolution Q3: Unit  DP -40.00  CXP -15.00  CE -27.00  IS: -4000.00  TEM: 600.00  GS1: 60.00  GS2: 55.00  CAD: 12.00 | |
| **Injection volume** | 1.0 µL | |

MRM Transitions:

| **Compound ID** | Mode | Q1 | Q3 | DP | CE | CXP |
| --- | --- | --- | --- | --- | --- | --- |
| **durlobactam** | Negative | 276.10 | 96.1 | -40 | -27 | -15 |
| **sulbactam** | Negative | 232.1 | 139.9 | -58 | -18 | -19 |
| **meropenem** | Positive | 384.1 | 141.0 | 25 | 21 | 10 |
| **Carbutamide (IS)** | Negative | 270.00 | 171.00 | -55 | -25 | -10 |

**Supplemental Information Table 4. Murine population PK Parameter Estimates for Sulbactam and Durlobactam**

| **Parameter^a,b^** | **Sulbactam Alone** | **Sulbactam In presence of durlobactam** | **Durlobactam in presence of sulbactam** |
| --- | --- | --- | --- |
| Ka (1/hr) | 6 (12.5) | 12 (8.4) | 13.5 (32.6) |
| V1 (L/kg) | 1.8847 | 1.0177 (7.0) | 1.2508 (8.4) |
| V2 (L/kg) | 0.57845 | 0.1332 (12.4) | 0.2282 (14.5) |
| Cl_1_ (L/kg/h) | 4.4514 | 2.5617 (5.2) | 3.0747 (7.3) |
| Cl_2_ (L/kg/h) | 0.09736 | 0.0299 (11.0) | 0.0641 (14.8) |
| Std Dev^c^ |  | 0.639 (4.9) | 0.554 (4.4) |
| Ω_Cl_ |  | 0.019 | 0.014 |
| Ω_V2_ |  | 0.204 | 0.052 |

^a^ predicted value (% coefficient of variance)

^b^ Ka = absorption rate V1 = central compartment volume of distribution, V2 = peripheral compartment volume of distribution, CL1 = clearance of central compartment CL2 = clearance of peripheral compartment

^c^ Standard Deviation refers to the standard deviation of Epsilon

**Supplemental Information Table 5. LC/MS/MS Assay Performance Summary**

| **Compound ID** | **LLOQ**  **(ng/mL)** | **ULOQ**  **(ng/mL)** | **Standards**  **% recovery (SD)** | **QC**  **% recovery (SD)** |
| --- | --- | --- | --- | --- |
| durlobactam | 1.0 | 10000 | 96.4% (10.1%) | 94.5% (8.3%) |
| sulbactam | 1.0 | 10000 | 99.2% (12.6%) | 98.9% (11.4%) |
| meropenem | 1.0 | 10000 | 96.3% (11.5%) | 98.5% (10.6%) |

LLOQ = lower limit of quantitation

ULOQ = upper limit of quantitation

SD = standard deviation

**Supplemental Figure 1. Change in bacterial burden (log10 CFU/g) over 24 h vs. unbound %fT>MIC of meropenem when administered in (A) neutropenic thigh model and in (B) neutropenic lung model vs. *A. baumannii* ARC2058.**

1. **(B)**


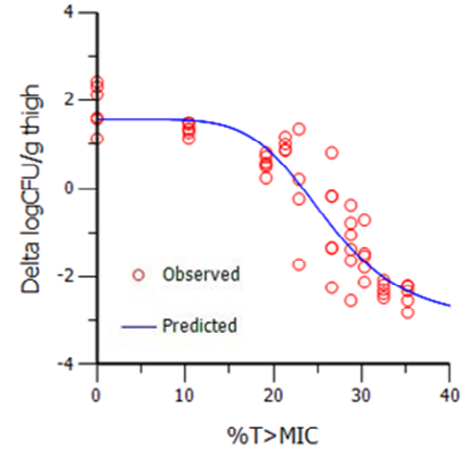

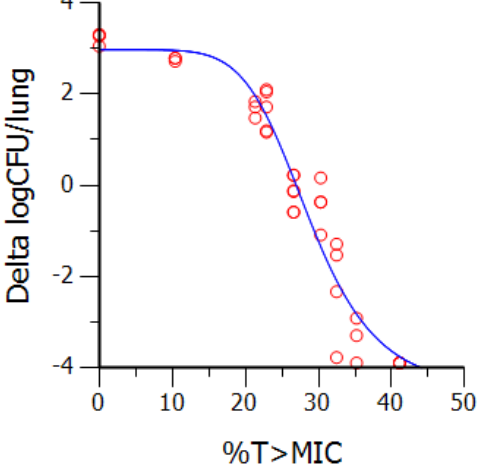


**Supplemental Figure 2. Two-compartment population PK model fitting of observed sulbactam and durlobactam concentration vs. time data following subcutaneous administration at a 4:1 dose ratio**


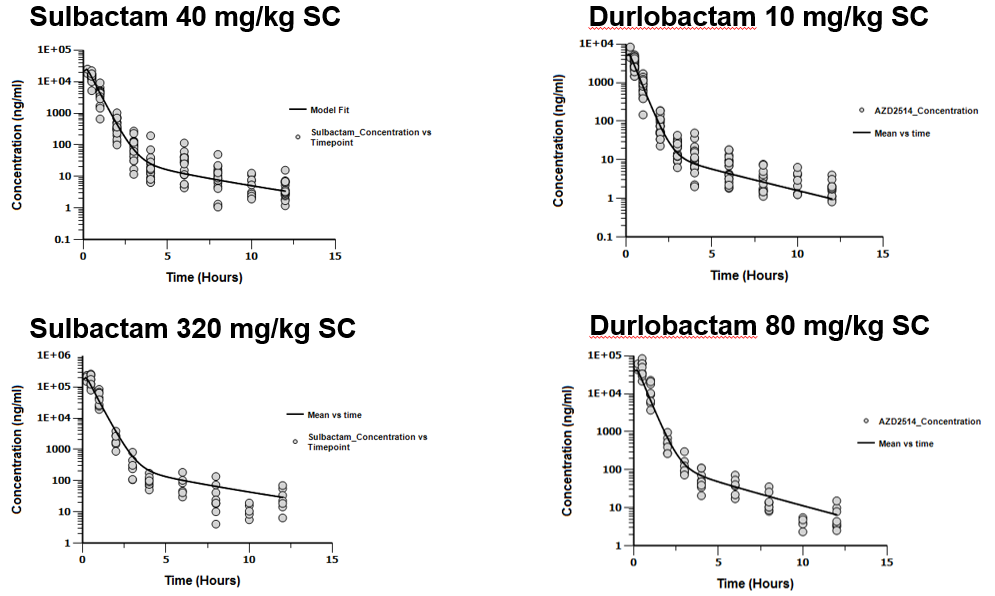


**AZD2514 = durlobactam**
